# Trend in body mass index during childhood in 460 girls with idiopathic central precocious puberty

**DOI:** 10.1530/EC-25-0215

**Published:** 2025-10-03

**Authors:** Alfredo Vicinanza, Obsse Oli Atomssa, Andrea Nebbioso, Fiorenza Ulgiati, Sophie Lambert, Sylvie Tenoutasse, Emese Boros, Claudine Heinrichs, Cécile Brachet

**Affiliations:** ^1^ Université libre de Bruxelles (ULB), Hôpital Universitaire de Bruxelles (H.U.B), Hôpital Universitaire des Enfants Reine Fabiola (HUDERF), Clinique d’endocrinologie pédiatrique, Bruxelles, Belgium; ^2^ Université libre de Bruxelles (ULB), Hôpital Universitaire de Bruxelles (H.U.B), Hôpital Universitaire des Enfants Reine Fabiola (HUDERF), Département de Pédiatrie, Bruxelles, Belgium; ^3^ Université libre de Bruxelles (ULB), Hôpital Etterbeek-Ixelles IRIS-Sud, Département de Pédiatrie, Bruxelles, Belgium

**Keywords:** BMI, idiopathic central precocious puberty, girls, childhood, adoption, migration

## Abstract

**Objective:**

A secular trend toward earlier puberty onset in girls has been widely documented, with childhood overweight proposed as a contributing risk factor. This study aims to characterize body mass index (BMI) standard deviation score (SDS) trajectories over the 6 years preceding idiopathic central precocious puberty (CPP) onset in girls.

**Design and methods:**

This retrospective, single-center study included 460 girls diagnosed with idiopathic CPP at the Academic Children's Hospital Queen Fabiola between 2002 and 2022. The cohort was stratified into sporadic CPP, familial CPP, and CPP in internationally adopted girls. Clinical and demographic data were collected, and BMI trajectories were analyzed using piecewise mixed linear models. Pubertal onset (T_0_) was defined as Tanner stage B2.

**Results:**

Among the 460 cases, 285 (62%) were sporadic, 145 (31.5%) familial, and 30 (6.5%) adoption-related CPP. In addition, 11.7% were born small for gestational age (SGA). BMI SDS increased significantly during the 6 years preceding T_0_ across the entire cohort. The steepest rise occurred between 6 and 3 years before T_0_ (+0.21 SDS/year (95% CI: 0.13–0.29)), followed by a slower increase in the 3 years before T_0_ (+0.15 SDS/year (95% CI: 0.11–0.19)), and a subsequent stabilization post-T_0_ (+0.06 SDS/year (95% CI: −0.01–0.14)). The BMI increase rate was similar across all subgroups.

**Conclusions:**

Girls with idiopathic CPP show a significant prepubertal BMI SDS increase, with similar trajectories in sporadic and familial cases. The overrepresentation of SGA-born and adopted girls suggests that genetic and environmental factors may contribute to early pubertal onset.

**Plain language summary:**

This study is the first to track BMI trajectories up to 6 years before idiopathic CPP onset in girls, revealing an early rise in BMI SDS across all subgroups (sporadic, familial, and adopted girls). Notably, SGA-born and adopted girls showed similar BMI patterns but were overrepresented in this CPP cohort.

## Introduction

Central precocious puberty (CPP) is characterized by the premature reactivation of the hypothalamic–pituitary–gonadal axis, occurring before the age of 8 in girls (1, 2, 3, 4, 5). The estimated prevalence of CPP ranges from 1:5,000 to 1:10,000 among Caucasian populations (6, 7, 8, 9). A significantly higher incidence in females compared to males (with a sex ratio of approximately 10:1) has been consistently reported worldwide (6, 7, 8, 9).

Over the centuries, a progressive decline in the age of pubertal onset has been observed, primarily attributed to improvements in socio-economic conditions and hygiene (10). More recently, this trend has persisted, as demonstrated by a systematic review by Eckert-Lind *et al.*, which reported a decrease of 0.24 years per decade in the age at thelarche between 1977 and 2013 (11). Notably, an alarmingly high incidence, along with an increasing trend in the prevalence of CPP, was documented in Korea between 2008 and 2014 (12). Similarly, in Denmark, Brauner *et al.* reported a six-fold increase in the annual incidence of CPP between 1998 and 2017 (13).

The variation in pubertal timing and progression is likely influenced by both genetic and environmental factors, including ethnicity, genetic predisposition, nutritional status, comorbid conditions, pharmacological exposures, physical activity levels, socio-economic status, and exposure to endocrine-disrupting chemicals, among other potential contributors.

Familial CPP is supposed to be associated with a genetic predisposition, whether monogenic (*MKRN3, DLK1*) or polygenic, although no genetic etiology could be elucidated in maternally transmitted CPP (14, 15, 16). Conversely, sporadic CPP is more frequently linked to environmental influences such as dietary factors, migration, and exposure to endocrine disruptors (16). Notably, several studies suggest that rapid weight gain during infancy and childhood may contribute to an earlier pubertal onset (16, 17, 18, 19). Moreover, persistent overweight and obesity in early childhood have been identified as significant risk factors for CPP in girls (20).

In addition, there is broad consensus that children born small for gestational age (SGA) tend to experience earlier pubertal onset compared to those born with an appropriate weight or length for gestational age (AGA) (21).

De Zegher *et al.* have proposed that precocious puberty may serve as a homeostatic response to excessive weight gain, conceptualizing a mismatch between pre- and postnatal weight trajectories. They hypothesize that an accelerated pubertal growth spurt may mitigate the risk of ectopic (hepato-visceral) adiposity (22). This hypothesis is supported by the study of Harbulot *et al.* (4), which showed that girls with sporadic CPP (presumably of environmental and metabolic origin) exhibited a greater discrepancy between birth weight standard deviation scores (SDS) and body mass index (BMI) SDS at the time of CPP diagnosis compared to those with familial CPP. Furthermore, Teilmann *et al.* observed an increased risk of CPP among adoptees, particularly those adopted after the age of 2 years, whereas children who migrated with their biological families did not exhibit a heightened risk of CPP (23).

The present study aims to validate these hypotheses within our cohort of girls diagnosed with idiopathic CPP. Specifically, we seek to analyze the trajectory of BMI SDS over the 6 years preceding CPP onset, comparing sporadic, familial, and adoption-related cases. In addition, we investigate the potential roles of origin, migration history, and auxological parameters at birth in influencing the timing of pubertal development.

## Materials and methods

### Patient selection and study design

We conducted a retrospective, single-center study, including girls diagnosed with idiopathic CPP who presented between January 2002 and December 2022 at the Paediatric Endocrinology Unit of the Academic Children's Hospital Queen Fabiola (HUDERF) in Brussels. Inclusion criteria comprised girls with a chronological age of <8 years at the onset of Tanner stage B2, increased height velocity, advanced bone age, basal luteinizing hormone (LH) levels >0.3 IU/L, and/or GnRH-stimulated peak LH levels >5 IU/L, uterine length >35 mm on ultrasound, or menarche before 10 years (2). Patients with incomplete diagnostic records, isolated premature thelarche and pubarche, or CPP due to organic causes were excluded.

### Methods

Our population of idiopathic CPP cases was categorized into three groups: sporadic, familial, and adopted cases. Familial CPP was defined by the presence of menarche before age 10 in a female family member and/or a history of early puberty in a male family member. When feasible, patients with familial paternally inherited CPP were screened for pathogenic variants in maternally imprinted genes associated with CPP (*MKNR3, DLK1*). Familial CPP cases were further classified into four subgroups based on the affected family member: maternal lineage, paternal lineage, siblings, and both parents (16).

For each patient, we collected birth date, dates of diagnosis (defined as the date of the initial clinical examination in our institution), menarche, and Tanner stage B2 (T_0_). Pubertal stages at diagnosis were assessed using the Tanner method (24). Standardized observation and palpation methods were applied by trained and certified staff. If menarche was the presenting complaint and no exact date was available, the 15th day of the month was recorded as the menarche date. To consider the reported date of Tanner stage B2, both the month and the year had to be provided, with the 15th day of the month being used when an exact date was unavailable. In the absence of the Tanner B2 exact date, according to the Tanner stage at diagnosis, the following rule was applied to estimate some of the B2 dates: subtracting 2 years if Tanner stage B4 and 1 year if Tanner stage B3 at the time of diagnosis (24, 25).

Data from 96 patients were considered unreliable due to inconsistencies and were therefore excluded from the analyses.

Neonatal auxological data were collected, and z-scores adjusted for birth term were obtained using the UK WHO ‘British 1990 Growth Reference, reanalyzed in 2009’ (26). Patients with birth weight and/or length < −2 SDS were classified as SGA (27). Birth weight SDS was adjusted for gestational age using the World Health Organization Anthro Survey Analyzer and other relevant tools.

BMI and its SDS values were recorded for each patient up to 6 years before Tanner stage B2 (T_0_) and up to the time of diagnosis, ensuring a minimum interval of 3 months between consecutive measurements. Growth data were extracted from school health service records or well-child encounter booklets.

Migration status was classified according to generational status. First-generation migrants included individuals born outside Belgium or those who were adopted from abroad. Second-generation migrants were defined as individuals born in Belgium to at least one parent of foreign origin (born outside Belgium). Ethnic origin was determined based on self-reported parental country of origin and categorized as follows: Caucasian (Europe, North America, and Australia), Maghreb, Sub-Saharan Africa, Asia (including the Middle East), Latin America, and Mixed origin.

Maternal age at menarche and reported early puberty in the father were documented. Parental consanguinity was also recorded.

Comorbidities were classified into three categories: neurological (e.g., autism, epilepsy), endocrinological (e.g., congenital adrenal hyperplasia), and other conditions.

Time 0 (T_0_) was defined as the onset of Tanner stage B2.

### Statistical analyses

Birth auxological data are presented as medians and interquartile ranges (IQR, p25–p75). For continuous variables with normal distributions, we applied Student’s *t*-test; while non-normally distributed continuous variables were analyzed using the Mann–Whitney test for two-group comparisons and the Kruskal–Wallis test for comparisons involving more than two groups. Discrete variables were expressed as percentages and analyzed using Chi-square tests.

To model the evolution of BMI SDS over time, we fitted two mixed linear regression models, considering Tanner stage B2 as time 0 (T_0_). We compared a simple linear mixed regression model with a piecewise linear mixed model incorporating two knots (one at T_0_ and another 3 years prior) using a likelihood ratio test. Both models included random effects on the intercept and slope coefficients without specific covariance structure assumptions. As the piecewise linear mixed model demonstrated superior fit, we subsequently fitted three separate piecewise mixed linear models, each incorporating a single time-interacting covariate: 1) type of CPP (sporadic, familial, or adopted), 2) migration status (none, first-generation, or second-generation), and 3) presence of SGA at birth.

All statistical analyses were conducted using STATA version 15.1 for MacOS (StataCorp, USA), with a significance threshold set at *P* < 0.05.

## Results

### Study population

**Figure 1:**
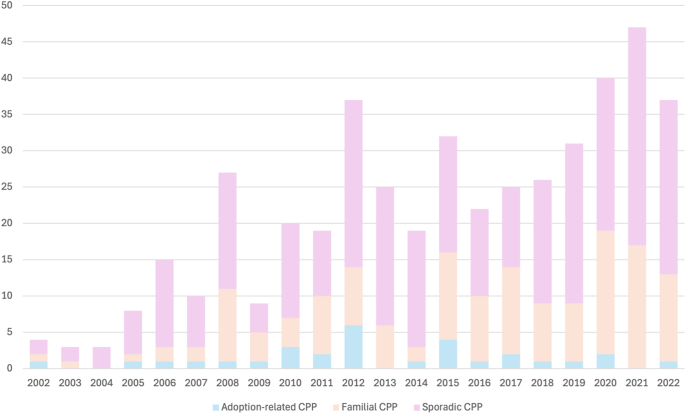
Total number of patients with idiopathic CPP per year in sporadic, familial, and adoption-related cases (*n* = 460).

We included 460 girls diagnosed with idiopathic CPP (Fig. 1), categorized into 285 (62%) sporadic cases, 145 (31.5%) familial cases, and 30 (6.5%) adopted cases.

### Cohort characteristics

The characteristics of the cohort are summarized in Table 1. The median age at T_0_ was 7.44 years. An associated Tanner stage *P* ≥ 2 (pubarche) was observed in 71% of patients at the time of diagnosis.

**Table 1:** Clinical characteristics of the population. Values are expressed as medians with IQR (p25–p75) or as *n* (%). *n* = number of patients for the corresponding characteristic.

Idiopathic CPP, *n* (%)		
* n* = 460	Sporadic	285 (61.96)
	Familial	145 (31.52)
	Adopted	30 (6.52)
Perinatal data		
* n* = 388	Weight at birth, median SDS (IQR)	−0.38 (−1.03–0.4)
* n* = 338	Length at birth, median SDS (IQR)	−0.16 (−0.97–0.53)
* n* = 342	SGA at birth, *n* (%)	40 (11.70)
Ages (years), median (IQR)		
* n* = 460	At diagnosis	8.25 (7.62–8.85)
* n* = 366	At T_0_ (Tanner B2)	7.44 (6.88–7.82)
* n* = 30	At adoption	4.00 (2.36–5.83)
Data at diagnosis, *n* (%)		
* n* = 460	BMI SDS ≥ 2	64 (13.91)
* n* = 445	Tanner *P* ≥ 2	314 (70.56)
* n* = 460	GnRH analogs treatment	343 (74.57)
Comorbidities, *n* (%)		
* n* = 460	Endocrinologic	12 (2.61)
	Neurologic	31 (6.73)
	Others	15 (4.17)
Origins, *n* (%)		
* n* = 424	Father	
	Caucasian	159 (37.5)
	Maghreb	60 (14.15)
	Sub-Saharan Africa	125 (29.48)
	Asia	61 (14.39)
	Latin America	15 (3.54)
	Mixed	4 (0.94)
* n* = 423	Mother	
	Caucasian	156 (36.88)
	Maghreb	52 (12.29)
	Sub-Saharan Africa	128 (30.26)
	Asia	63 (14.89)
	Latin America	20 (4.73)
	Mixed	4 (0.95)
Migration, *n* (%)		
* n* = 407	No migration	50 (12.29)
	1st generation migrants	88 (21.62)
	2nd generation migrants	269 (66.09)

Among the adopted children, the median age at adoption was 4 years (IQR: 2.36–5.83).

Comorbidities, primarily neurological (7%), were present in 12.6% of the cohort.

Most patients were of Caucasian or Sub-Saharan African origin. Second-generation immigrants represented the largest group (66%), compared to 12% of Belgian origin (i.e., no migration, neither of first nor of second generation). The median birth weight and length SDS were below the population standards, with 12% of the cohort classified as SGA at birth (Table 1).

Among the 145 familial cases, maternal transmission was more commonly reported than paternal transmission (42.1 vs 31.7%). Genetic analysis was conducted in 39 patients (37 families), revealing pathogenic *MKNR3* gene variants in 11 cases. Twenty-seven consanguineous families were identified (data not shown).

### Group comparisons

We compared clinical and demographic characteristics across the three CPP subgroups (sporadic, familial, and adopted; Table 2). No significant differences were observed between sporadic and familial CPP groups, except for comorbidities, which were more prevalent in sporadic CPP cases (*P* = 0.01).

**Table 2:** Comparison of clinical and demographic characteristics of the three groups of CPP (sporadic, familial, and adoption-related). Values are expressed as medians with IQR (p25–p75) or as *n* (%). *n* = number of patients for the corresponding characteristic. *P*-value 1, between sporadic and familial CPP; *P*-value 2, among sporadic, familial, and adoption-related CPP.

	*n*	Sporadic	*n*	Familial	*P*-value 1	*n*	Adopted	*P*-value 2
		*n* = 285; 62%		*n* = 145; 31.5%			*n* = 30; 6.5%	
Birth weight, SDS	251	−0.38 (−1.05–0.37)	133	−0.38 (−0.95–0.56)	0.47	4	−0.94 (−1.68–−0.19)	0.42
Birth length, SDS	214	−0.17 (−0.97–0.53)	121	−0.01 (−0.8–0.53)	0.63	3	−1.63 (−3.04–0.53)	0.40
SGA	218	27 (12.39)	121	12 (9.92)	0.49	3	1 (33.33)	0.4
Age at diagnosis, years	285	8.26 (7.61–8.85)	145	8.22 (7.67–8.73)	0.75	30	8.4 (7.62–8.84)	0.71
Age at B2 (T_0_), years	221	7.49 (6.86–7.85)	122	7.43 (6.97–7.78)	0.05	23	7.36 (6.65–7.92)	0.12
BMI at diagnosis, SDS ≥ 2	285	43 (15.1)	145	20 (13.8)	0.71	30	1 (3.33)	0.21
Comorbidities	285		145		0.013	30		0.012
Yes		46 (16.14)		11 (7.59)			1 (3.33)	
No		239 (83.86)		134 (92.41)			29 (96.67)	
Fathers’ origin	264		131		0.01	29		<0.01
Caucasian		101 (38.26)		55 (41.98)			3 (10.34)	
Maghreb		40 (15.15)		20 (15.27)			0	
Sub-Saharan Africa		84 (31.82)		33 (25.19)			8 (27.59)	
Asia		32 (12.12)		13 (9.92)			16 (55.17)	
Latin America		3 (1.14)		10 (7.63)			2 (6.9)	
Mixed		4 (1.52)		0			0	
Mothers’ origin	262		132		0.26	29		<0.01
Caucasian		97 (37.02)		56 (42.42)			3 (10.34)	
Maghreb		35 (13.36)		17 (12.88)			0	
Sub-Saharan Africa		86 (32.82)		34 (25.76)			8 (27.59)	
Asia		33 (12.6)		14 (10.61)			16 (55.17)	
Latin America		8 (3.05)		10 (7.58)			2 (6.9)	
Mixed		3 (1.15)		1 (0.76)			0	
Migration	249		128		0.14	30		<0.01
No migration		28 (11.24)		22 (17.19)			0	
1st generation		43 (17.27)		15 (11.72)			30 (100)	
2nd generation		178 (71.49)		91 (71.09)			0	

At the time of CPP diagnosis, obesity prevalence was 15% in sporadic cases, 14% in familial cases, and 3% among adopted girls (*P* = 0.21) (Table 2).

### BMI evolution over time

Table 3 presents the output of the models demonstrating BMI SDS evolution across different groups.

**Table 3:** Estimates of BMI SDS gain over time and BMI SDS at Tanner B2 (T_0_) obtained with four different piecewise mixed linear regression models.

Fixed effects	BMI SDS			
	Coefficient	*P*-value	(95% conf. interval)	
Overall population (model with no covariates)				
Intercept (SDS at Tanner B2)	0.78	<0.001	0.66	0.89
Year 4–6 before B2 (SDS gain per year)	0.21	<0.001	0.13	0.29
Year 0–3 before B2 (SDS gain per year)	0.15	<0.001	0.11	0.19
After B2 (SDS gain per year)	0.06	0.089	−0.01	0.14
* n* of patients	366			
* n* of observations	1,171			
Model with group of CPP as covariate				
Sporadic form				
Intercept (SDS at Tanner B2)	0.82	<0.001	0.67	0.97
Year 4–6 before B2 (SDS gain per year)	0.23	<0.001	0.13	0.38
Year 0–3 before B2 (SDS gain per year)	0.15	<0.001	0.10	0.21
After B2 (SDS gain per year)	0.08	0.107	−0.02	0.18
Familial form				
Intercept (SDS at Tanner B2)	0.81	<0.001	0.62	1.01
Year 4–6 before B2 (SDS gain per year)	0.15	0.028	0.02	0.29
Year 0–3 before B2 (SDS gain per year)	0.13	<0.001	0.06	0.20
After B2 (SDS gain per year)	0.05	0.435	−0.07	0.17
Adopted girls				
Intercept (SDS at Tanner B2)	0.22	0.311	−0.20	0.64
Year 4–6 before B2 (SDS gain per year)	0.37	0.032	0.03	0.71
Year 0–3 before B2 (SDS gain per year)	0.16	0.036	0.01	0.30
After B2 (SDS gain per year)	−0.07	0.659	−0.38	0.24
* n* of patients	366			
* n* of observations	1,171			
Model with SGA as covariate				
AGA at birth				
Intercept (SDS at Tanner B2)	0.83	<0.001	0.70	0.97
Year 4–6 before B2 (SDS gain per year)	0.18	<0.001	0.09	0.27
Year 0–3 before B2 (SDS gain per year)	0.14	<0.001	0.10	0.19
After B2 (SDS gain per year)	0.06	0.156	−0.02	0.15
SGA at birth				
Intercept (SDS at Tanner B2)	0.28	0.165	−0.11	0.67
Year 4–6 before B2 (SDS gain per year)	0.36	0.002	0.13	0.59
Year 0–3 before B2 (SDS gain per year)	0.15	0.027	0.02	0.28
After B2 (SDS gain per year)	0.13	0.386	−0.16	0.42
* n* of patients	279			
* n* of observations	931			
Model with migration as covariate				
No migration				
Intercept (SDS at Tanner B2)	0.52	0.001	0.20	0.85
Year 4–6 before B2 (SDS gain per year)	0.12	0.257	−0.09	0.33
Year 0–3 before B2 (SDS gain per year)	0.17	0.001	0.06	0.27
After B2 (SDS gain per year)	0.06	0.609	−0.17	0.29
Migration 1st generation				
Intercept (SDS at Tanner B2)	0.41	0.002	0.15	0.68
Year 4–6 before B2 (SDS gain per year)	0.39	0.002	0.15	0.64
Year 0–3 before B2 (SDS gain per year)	0.08	0.125	−0.02	0.19
After B2 (SDS gain per year)	0.17	0.067	−0.01	0.35
Migration 2nd generation				
Intercept (SDS at Tanner B2)	0.95	<0.001	0.80	1.09
Year 4–6 before B2 (SDS gain per year)	0.20	<0.001	0.10	0.30
Year 0–3 before B2 (SDS gain per year)	0.17	<0.001	0.12	0.22
After B2 (SDS gain per year)	0.05	0.293	−0.04	0.15
* n* of patients	326			
* n* of observations	1,056			

At T_0_, the overall BMI SDS was +0.78 (CI: +0.66, +0.89). The BMI SDS increase was most pronounced between 6 and 3 years before T_0_ (+0.21 SDS per year (CI: +0.13, +0.29)), followed by a slower increase during the 3 years preceding T_0_ (+0.15 SDS per year (CI: +0.11, +0.19)), and a plateau after T_0_ (+0.06 SDS per year (CI: −0.01, +0.14)).

**Figure 2:**
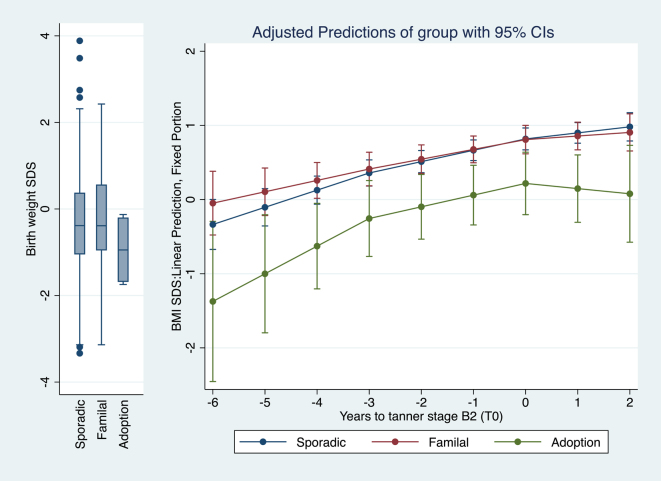
Piecewise mixed linear regression model estimation of BMI SDS over the six years preceding T_0_ according to groups (sporadic, familial, and adoption-related) (*n* = 366, *n* of observations = 1,171).

**Figure 3:**
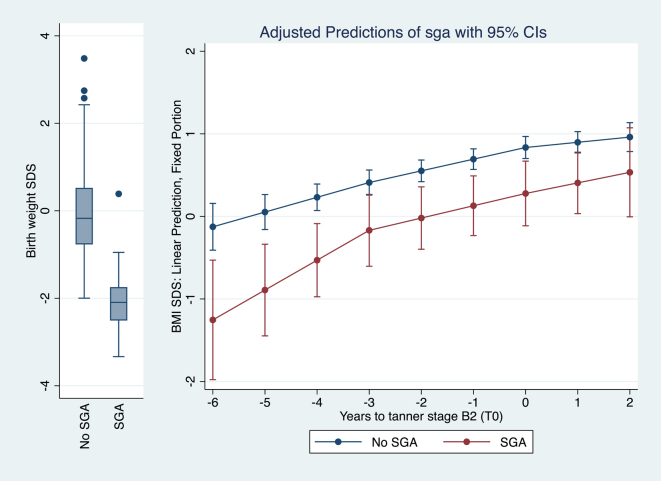
Piecewise mixed linear regression model estimation of BMI SDS over the six years preceding T_0_ according to SGA birth (*n* = 279, *n* of observations = 931).

**Figure 4:**
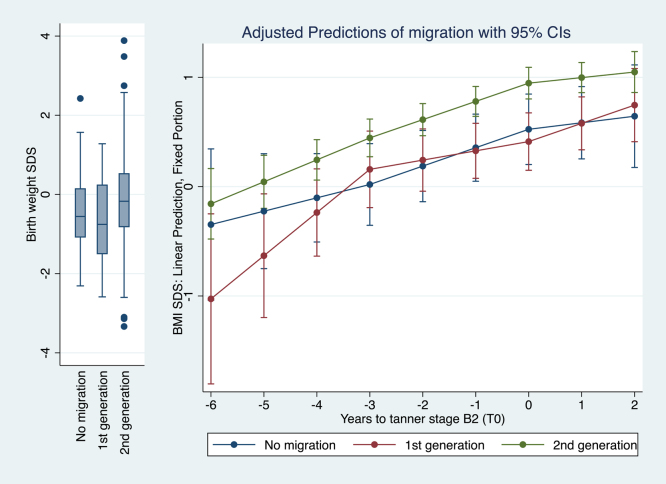
Piecewise mixed linear regression model estimation of BMI SDS over the six years preceding T_0_ according to migration (*n* = 326, *n* of observations = 1,056).

Figures 2, 3, 4 provide graphical representations of the models with covariates detailed in Table 3.

Over the 6 years preceding T_0_, a statistically significant increase in BMI SDS was observed across all CPP groups (Fig. 2). Adopted girls had a consistently lower BMI SDS throughout this period, with a BMI at T_0_ of +0.22 SDS (CI: −0.20, +0.64), compared to sporadic (+0.82 SDS (CI: +0.67, +0.97)) and familial cases (+0.81 SDS (CI: +0.62, +1.00)).

Similarly, BMI SDS increased significantly over the 6-year period in both SGA and non-SGA children, despite evidence of catch-up growth among SGA children from birth to the onset of CPP (data not shown). Nevertheless, SGA girls consistently exhibited lower BMI SDS values compared to their non-SGA counterparts throughout the years preceding and up to the time of CPP diagnosis. At T_0_, the mean BMI SDS for SGA girls was +0.28 (95% CI: −0.11, +0.67), whereas non-SGA girls had a mean BMI SDS of +0.83 (95% CI: +0.70, +0.97) (Fig. 3 and Table 3).

Regardless of BMI category at diagnosis (normal-weight or overweight/obese patients), the trajectory of BMI over time is similar across groups, differing only in absolute BMI values (overweight/obese patients maintaining a higher BMI throughout), not in trend (data not shown).

Among migration groups, second-generation migrants exhibited a significant BMI SDS increase over the entire 6-year period before T_0_ (Fig. 4). In contrast, first-generation migrants showed significant BMI SDS increases only between 6 and 3 years before T_0_, and non-migrants between 3 years before T_0_ and T_0_ (Fig. 4). Second-generation migrants had the highest BMI SDS at T_0_ (+0.95 SDS (CI: +0.80, +1.09)), compared to non-migrants (+0.52 SDS (CI: +0.20, +0.85)) and first-generation migrants (+0.41 SDS (CI: +0.15, +0.68)).

Notably, BMI SDS increase was no longer significant after T_0_ across all groups. Furthermore, no statistically significant differences were found in BMI SDS slope (gain/year) among the three CPP subgroups, migration groups, or between SGA and AGA patients.

The difference between the median birth weight SDS and BMI SDS at T_0_ did not differ significantly among the three groups (sporadic, familial, and adopted) (data not shown).

Moreover, analyses restricted to CPP patients without comorbidities demonstrate subgroup patterns consistent with those reported earlier in the present study (data not shown).

The majority of patients (75%) received GnRH analogs (GnRHa) to slow down pubertal progression, although a trend toward reduced treatment utilization has been observed in recent years (data not shown).

## Discussion

This single-center retrospective study is the first to document BMI trends up to 6 years before pubertal onset, defined as Tanner stage B2 (T_0_), in a cohort of girls diagnosed with idiopathic CPP. Understanding these BMI trajectories may provide insight into the interplay between genetic, environmental, and metabolic factors influencing early puberty.

Among the 460 girls included, 31% were classified as familial CPP, a prevalence consistent with findings from Durand *et al.* (28). Notably, maternal transmission was predominant, in line with previous studies (28). However, research by Harbulot and Tinano (4, 16) reported similar rates of maternal and paternal inheritance. This discrepancy may arise from differential recall accuracy, as women typically remember their age at menarche more precisely than men recall their pubertal timing (16, 29). Furthermore, paternal inheritance may have been underreported, particularly before 2013, when pathogenic variants in paternally transmitted genes such as *MKRN3* had yet to be identified (16, 30). These findings highlight the complexities of genetic contributions to CPP and the challenges in accurately documenting inheritance patterns.

In our cohort, 11.7% of patients were born SGA, significantly exceeding the estimated 2.5% prevalence in the general population (27). Low birth weight has been consistently identified as a risk factor for both CPP and early puberty (21, 31). Studies indicate that girls born SGA or with low birth weight tend to experience earlier pubertal onset than their appropriate-for-gestational-age (AGA) counterparts, although statistical significance has primarily been established in girls born light for gestational age (32). Lazar *et al.* reported that early puberty occurred in 20% of SGA girls and 13% of SGA boys, compared to 3 and 5% of their AGA peers, respectively (33). These findings suggest a biological predisposition linking intrauterine growth restriction to altered pubertal timing.

Adopted girls represented 6.5% of our cohort, with a median adoption age of 4 years. This overrepresentation suggests a potential association between adoption and early puberty. Similar findings have been reported in prior studies, including Proos *et al.*’s 1991 analysis of 107 Swedish-adopted Indian girls, who experienced menarche significantly earlier than both Swedish and Indian non-adopted populations (34). Subsequent research across Western Europe has corroborated this trend (35). In 2006, Teilmann *et al.* quantified the risk, demonstrating that internationally adopted children, particularly those adopted after age two, had a 15- to 20-fold increased likelihood of developing CPP compared to Danish-born children (23). Although the causal link between adoption and CPP remains debated, several hypotheses have been proposed, focusing on the impact of emotional and environmental factors. Potential mechanisms include intrauterine growth restriction, early-life nutritional deficits, post-adoption catch-up growth leading to increased adiposity, and psychosocial stress, all of which may contribute to early puberty (10, 36).

Across the entire cohort, BMI SDS exhibited a progressive increase in the 6 years preceding T_0_, with the most pronounced gain occurring between 6 and 3 years prior (+0.21 SDS/year). This increase slowed in the 3 years immediately before T_0_ (+0.15 SDS/year) and plateaued thereafter.

T_0_ corresponds to Tanner stage B2. The observed steep increase in BMI SDS between 6 and 3 years before T_0_ occurs notably earlier than the physiological adiposity rebound (AR), which generally takes place between 5 and 7 years of age (37). This early BMI increase may reflect a precocious AR, characterized by rapid metabolic and hormonal changes that predispose to earlier pubertal onset. Our findings support the hypothesis that puberty is programmed by BMI in infancy, consistent with the life–history transitions theory proposed by German *et al.* and Hochberg *et al.* (38, 39).

A precocious AR could predispose to CPP through complex neuroendocrine and metabolic interactions. Excess adiposity during this critical window presumably elevates circulating leptin and insulin levels (15, 40, 41). Leptin and insulin act as key metabolic signals to the hypothalamus by enhancing pro-opiomelanocortin (POMC) and suppressing neuropeptide Y (NPY) transcription. The Kisspeptin–Neurokinin B–Dynorphin A (KNDy) neurons in the arcuate nucleus harbor insulin and leptin receptors. Furthermore, cellular energy sensors such as mTOR, Sirt1, and AMP-activated protein kinase link energy status to pubertal activation at the level of POMC/NPY and KNDy neurons. Other repressor elements, such as DLK1, also display impaired function in obesity, suggesting additional mechanisms connecting adiposity to pubertal onset (40, 41, 42, 43, 44). In addition, altered adipokine profiles and low-grade inflammation associated with early adiposity may disrupt hypothalamic regulation via cytokines such as TNF-α and IL-6, further accelerating pubertal timing (42, 43).

The secular trend toward earlier puberty in girls has been hypothesized to correlate with the rising prevalence of childhood obesity (45, 46). Longitudinal studies consistently demonstrate that overweight girls experience earlier pubertal development (19, 47), and cross-sectional studies report a significant association between female obesity and early menarche, accelerated growth velocity, and premature pubarche (47). A 2021 case–control study by Liu *et al.* further established an independent association between overweight/obesity and CPP in girls, controlling for birth characteristics, diet, and socioeconomic factors (20).

At the time of CPP diagnosis, the rate of obesity was comparable between the familial and sporadic cases in our cohort and notably higher than the national reference data. Specifically, obesity was observed in 15% of girls with sporadic CPP and 14% of those with familial CPP. According to the 2022–2023 National Dietary Consumption Survey, the prevalence of obesity in the general Belgian population increases progressively with age, reported at 4% in children, 7% in adolescents, and peaking at 28% in individuals aged 65 years and older (48). However, our data do not account for pubertal status based on Tanner staging at the time of CPP diagnosis, which can significantly affect BMI interpretation. Since BMI SDS reference curves are derived from populations with average pubertal timing, and puberty naturally involves an increase in both fat and lean mass (49), the earlier onset in CPP may partly explain the elevated BMI in our cohort. Nonetheless, BMI trajectories over time were nearly parallel across BMI categories at diagnosis (normal weight vs overweight/obese) and across subgroups (familial, sporadic, adopted), with overweight/obese patients consistently showing higher absolute BMI values but similar progression patterns to normal-weight girls (data not shown).

The subsequent stabilization of BMI SDS post-T_0_ is a surprising finding because pubertal development goes along with an increase in BMI. This plateau may be partially explained by the clinical follow-up, which usually involves dietary and lifestyle counseling. However, GnRHa probably has a modest short-term effect on BMI, as supported by various studies and highlighted in a recent meta-analysis reporting an increase in BMI SDS between the start and the end of GnRHa treatment in girls with CPP (weighted mean difference = +0.15; 95% CI: +0.05, +0.25) (50, 51).

Despite variations in absolute BMI levels, the rate of BMI increase was similar across subgroups. Moreover, differences in birth weight and BMI SDS at diagnosis were not significant across all three groups (sporadic, familial, and adopted), challenging de Zegher’s empiric mismatch hypothesis (22), which suggests that sporadic CPP cases should exhibit a more pronounced prepubertal BMI increase than familial or adoption-related cases. However, the distinction between familial and sporadic CPP is often ambiguous, potentially limiting the robustness of this analysis.

A similar BMI trajectory was observed among SGA- and AGA-born girls. Previous research studies suggest that SGA girls undergo rapid weight gain in early childhood and face an elevated risk of metabolic syndrome in adulthood (52). In addition, SGA children who experience catch-up growth before age two tend to have a higher BMI and greater central fat distribution by age five compared to their peers (53). This pattern has been linked to long-term metabolic programming, yet in our cohort, BMI progression up to 6 years before CPP onset in girls born SGA closely mirrored that of non-SGA girls.

BMI trajectories varied by migration status. Non-migrant girls exhibited an increase in BMI between 6 and 3 years before T_0_, whereas first-generation migrants only showed BMI increase in the 3 years preceding puberty. In contrast, second-generation migrants demonstrated a continuous BMI increase. The mechanisms underlying these differences remain unclear but may relate to early-life environmental transitions, as observed in adoptees (54). While an increased CPP risk has been well documented in internationally adopted children, no such association has been established in children migrating with their families (35, 54).

### Strengths and limitations

A key strength of this study lies in its large, well-characterized cohort, enabling comparison among sporadic, familial, and adopted cases. The single-center design ensured standardized clinical assessments and high-quality longitudinal BMI data, enhancing the reliability of findings. However, the retrospective nature introduces recall bias, particularly concerning age at B2 onset, menarche, and paternal pubertal timing. In addition, familial cases may have been underreported due to incomplete or unavailable parental histories. Data on adopted girls were also limited, restricting the interpretability of findings within this subgroup. Moreover, the retrospective design of the study constrains the possibility of incorporating other potentially confounding variables, such as parental BMI, dietary habits, and lifestyle factors, into the longitudinal analysis.

The absence of a control group prevents definitive conclusions regarding whether the observed BMI increase reflects a general trend of rising childhood overweight or constitutes a specific risk factor for CPP, or an early metabolic adaptation preceding the onset of CPP. Future research incorporating appropriate control groups is necessary to clarify this relationship.

## Conclusion

This study documents a significant increase in BMI during the 6 years preceding the onset of idiopathic CPP, consistent across all subgroups, suggesting that puberty may be programmed by BMI in infancy. We found no evidence that sporadic CPP cases exhibit a more pronounced BMI increase than familial CPP cases. The overrepresentation of SGA-born and adopted girls underscores the complex interplay of genetic, epigenetic, and environmental factors in pubertal timing. Further research is warranted to elucidate these mechanisms and refine our understanding of the link between BMI and pubertal onset.

## Declaration of interest

The authors declare that there is no conflict of interest that could be perceived as prejudicing the impartiality of the work reported.

## Funding

This work did not receive any specific grant from any funding agency in the public, commercial, or not-for-profit sector.

## Author contribution statement

AV and CB conceived and designed the study. AV and OOA drafted, wrote, and structured the manuscript and were primarily responsible for data acquisition, analysis, and interpretation. AN contributed to statistical analysis, graphics, and the interpretation of results. FU, SL, and ST assisted with data acquisition and contributed to the interpretation of results. AV, CH, EB, and CB substantially contributed to the study’s conception and design, as well as data acquisition, analysis, and interpretation, and critically revised the manuscript for important intellectual content. All authors participated in data interpretation, patient management (except for AN and OOA), and manuscript development. All authors critically reviewed the manuscript and approved the final version for publication.

## Data availability

The original contributions presented in this study are included in the article/supplementary material. Further inquiries can be directed to the corresponding author.

## Ethical declaration

The study protocol was approved by the ethics committee of HUDERF (CEH no. 49/23), and all reported investigations were conducted in accordance with the principles of the Declaration of Helsinki, as revised in 2013.
